# Supplementary figures and images for: Integrated Multi-omics Investigations Reveal the Key Role of Synergistic Microbial Networks in Removing Plasticizer Di-(2-Ethylhexyl) Phthalate from Estuarine Sediments
Source: mSystems. 2021 Jun 8;6(3):e00358-21. doi: 10.1128/mSystems.00358-21 (PMC8269228; doi:10.1128/mSystems.00358-21)

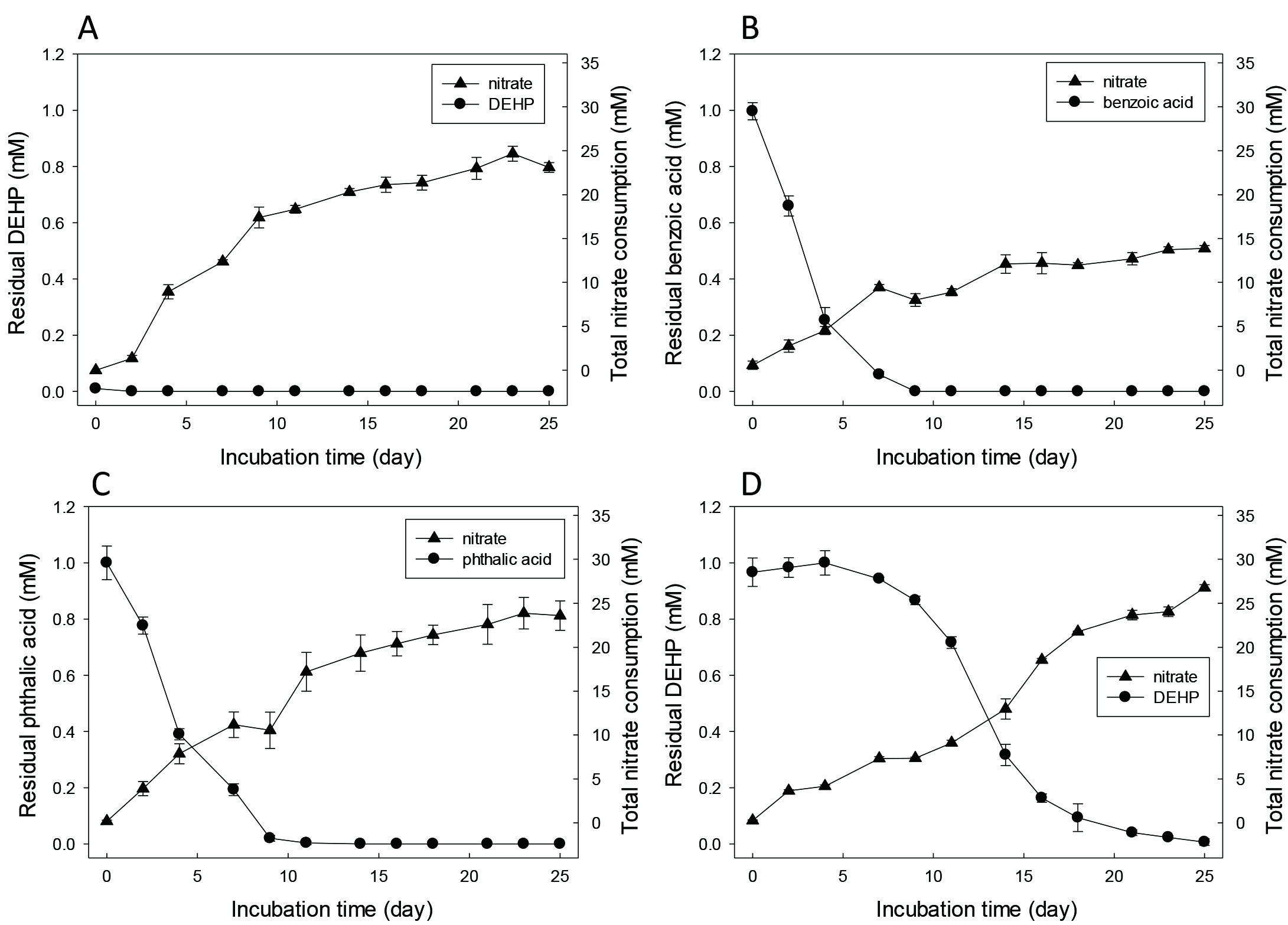

Supplement: FIG S1 [file msystems.00358-21-sf001.jpg]

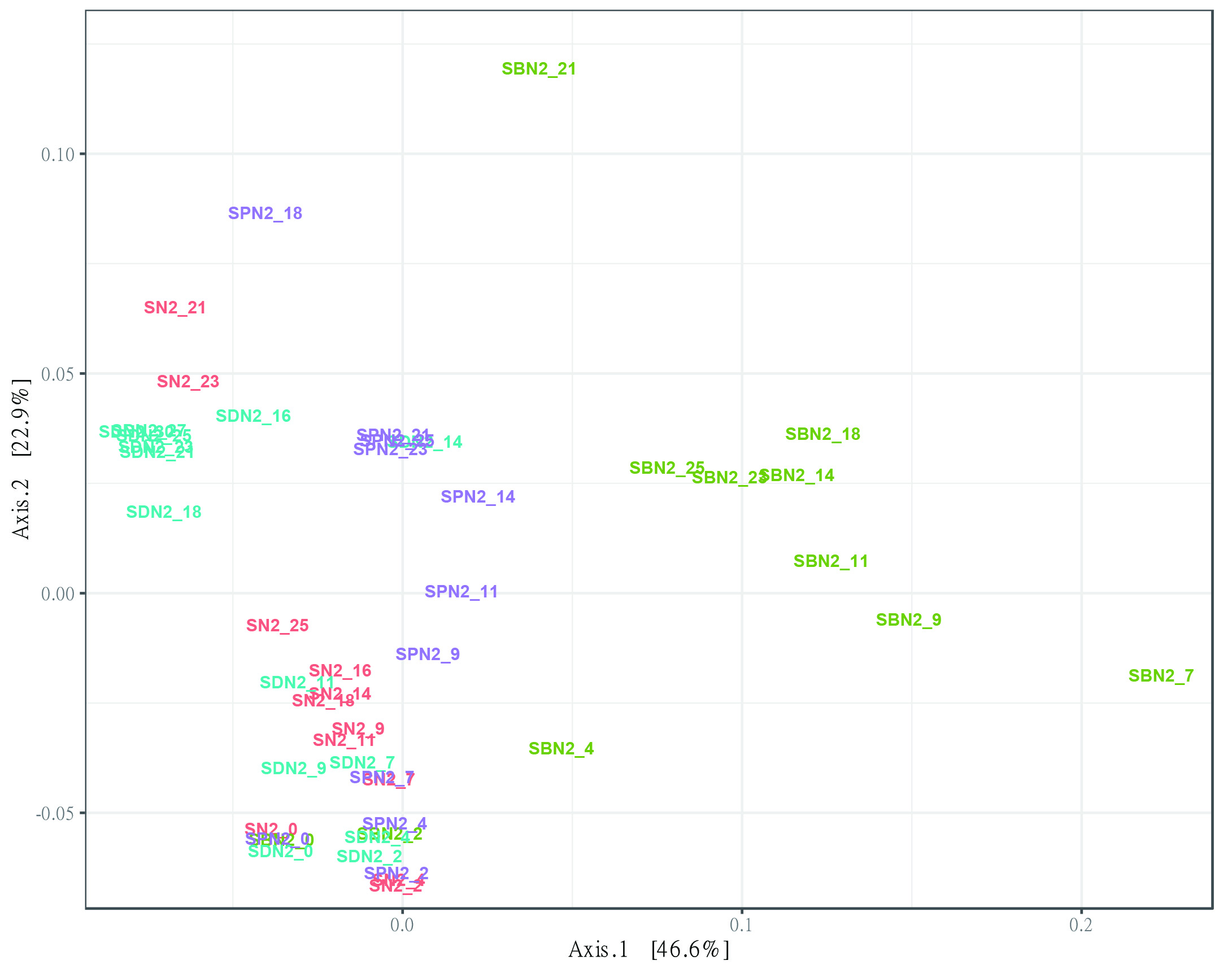

Supplement: FIG S2 [file msystems.00358-21-sf002.jpg]

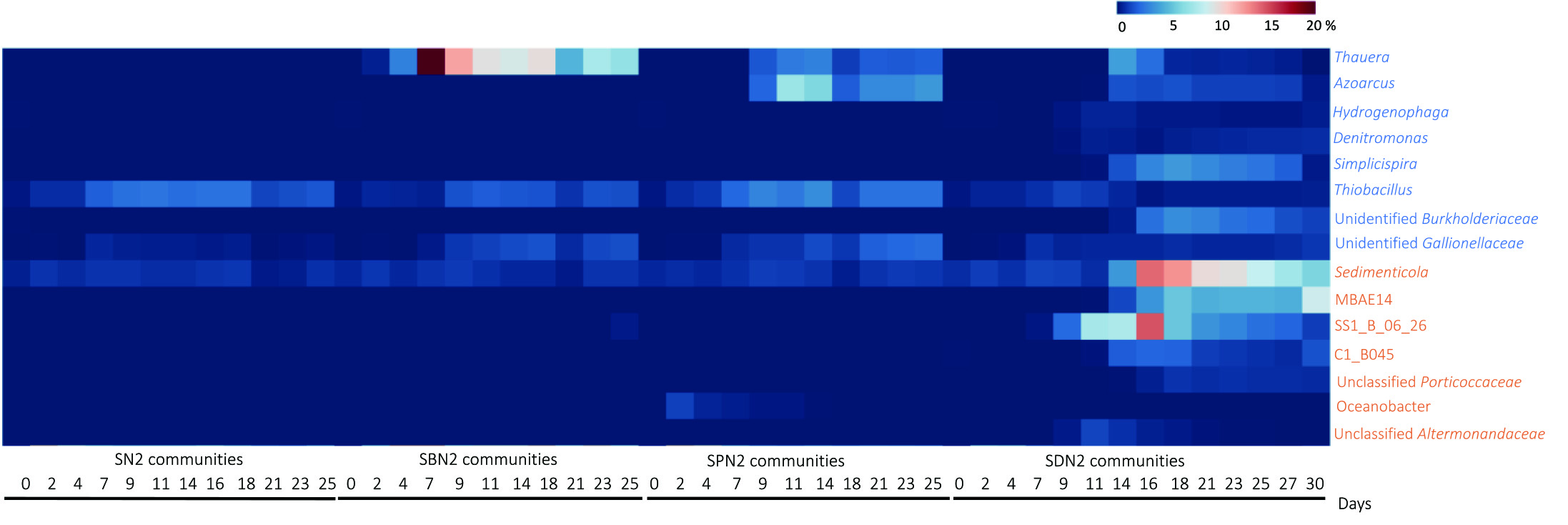

Supplement: FIG S3 [file msystems.00358-21-sf003.jpg]

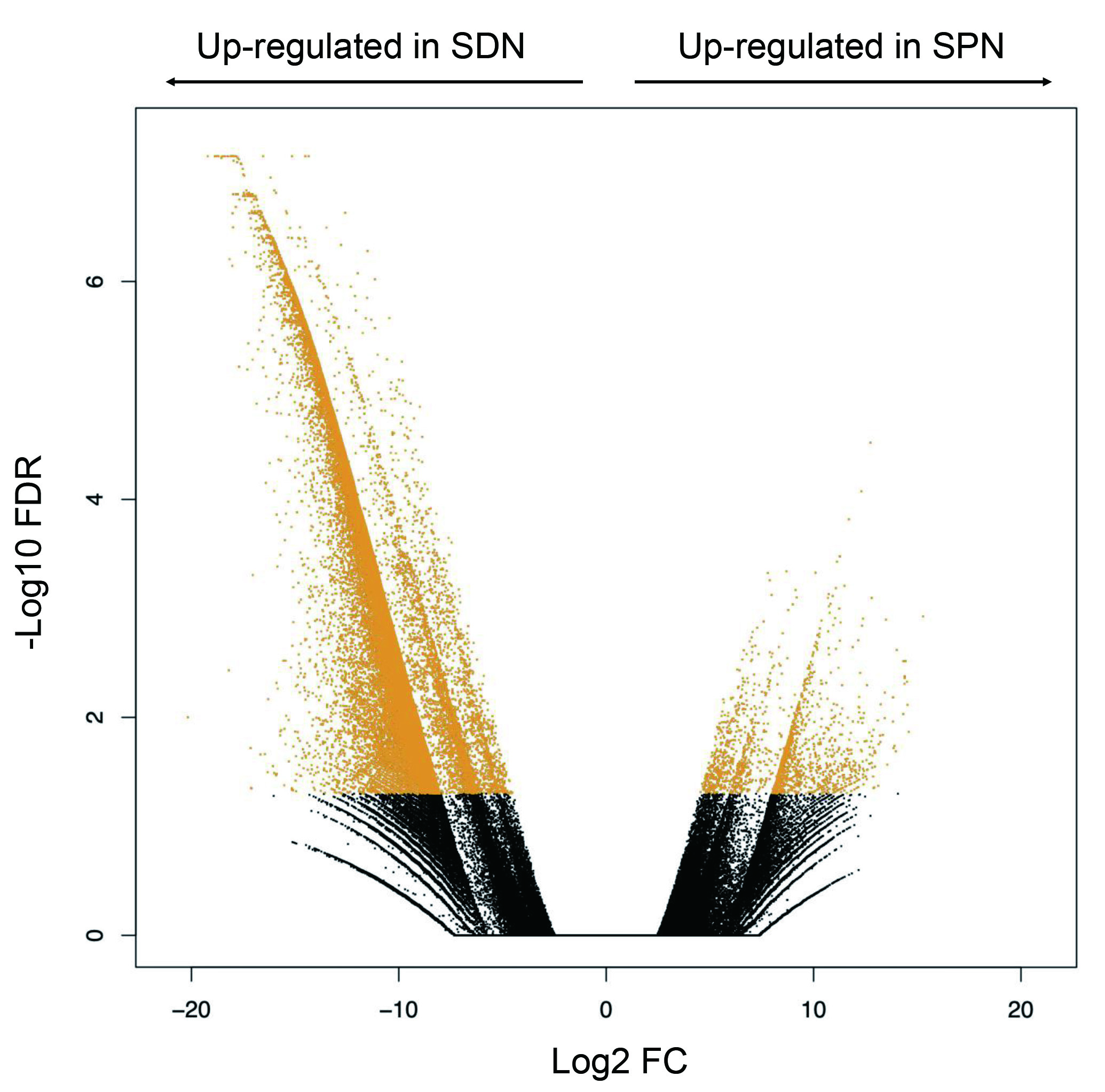

Supplement: FIG S4 [file msystems.00358-21-sf004.jpg]

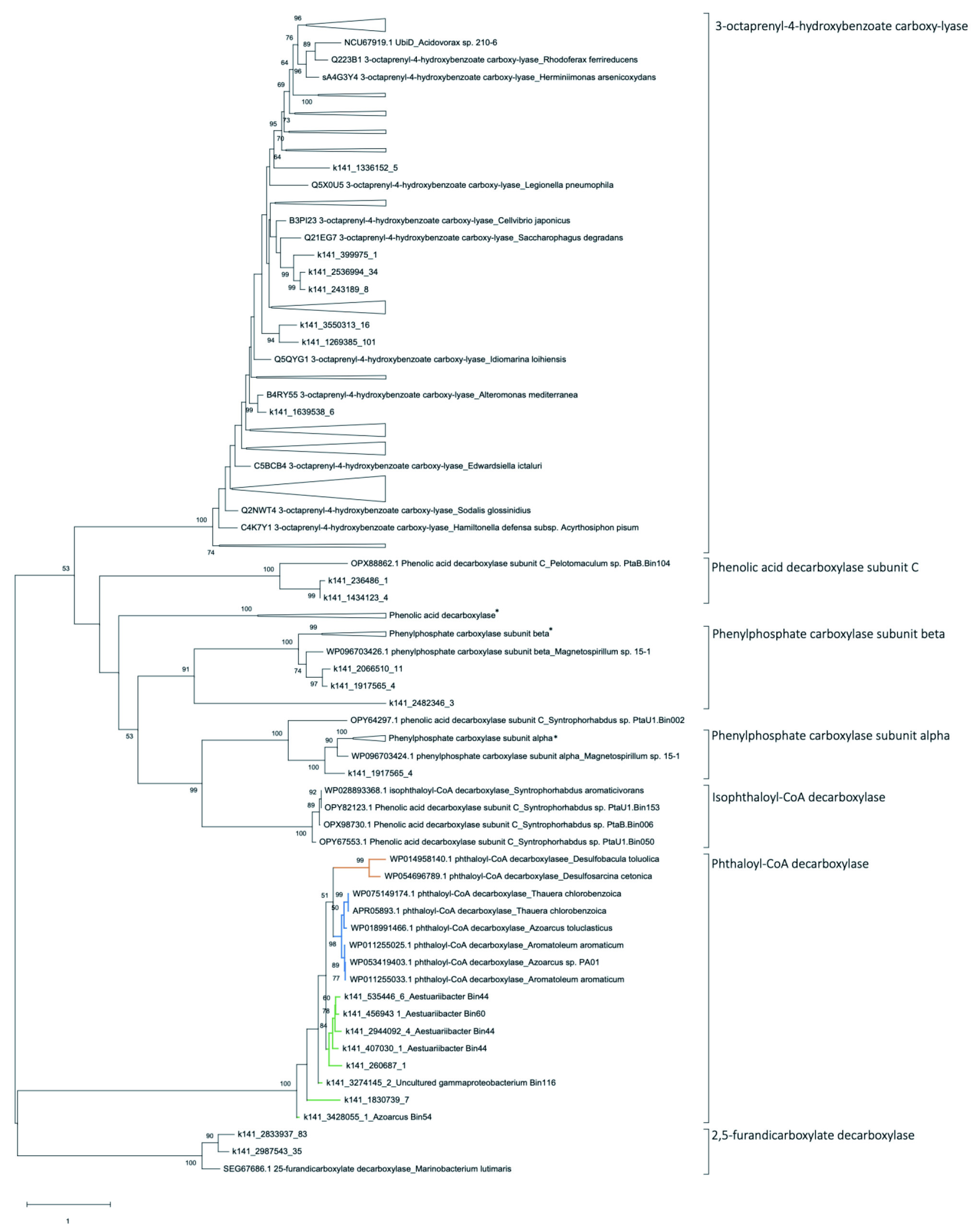

Supplement: FIG S5 [file msystems.00358-21-sf005.jpg]
