# Supplementary material for: Integrated Multi-omics Investigations Reveal the Key Role of Synergistic Microbial Networks in Removing Plasticizer Di-(2-Ethylhexyl) Phthalate from Estuarine Sediments
Source: mSystems. 2021 Jun 8;6(3):e00358-21. doi: 10.1128/mSystems.00358-21 (PMC8269228; doi:10.1128/mSystems.00358-21)
Supplement: TEXT S1 [file msystems.00358-21-t0001.pdf]

Integrated multi-omics investigations reveal the important role of synergistic microbial metabolism in removing plasticizer di-(2-ethylhexyl) phthalate from estuarine sediments

Running title: microbial plasticizer degradation in sediments

Sean Ting-Shyang Wei, Yi-Lung Chen, Yu-Wei Wu, Po-Hsiang Wang, Yi-Li Lai, Tien-Yu-Wu, Wael Ismail, Tzong-Huei Lee, and Yin-Ru Chiang

Supplemental Information (SI) containing SI Results and SI References

Supplemental Results

Binned genomes with complete *o*-phthalic acid degradation capacity in the *o*-phthalic acid-treated mesocosms. Among the 1800 up-regulated genes in the *o*-phthalic acid-treated mesocosms, we identified some genes involved in the uptake and degradation of *o*-phthalic acid. For example, a *o*-phthalic acid transporter gene (k141_3776202_21) was identified in Bin97. The protein encoded by this gene displayed closest amino acid identify (AAI) to the corresponding protein from *Thauera aromatica*. The Bin97 also contained fused permease of *o*-phthalic acid transporter gene (k141_3776202_22). On the other hand, among the 1800 up-regulated genes, we observed two CoA transferase genes (k141_1104744_2 and k141_1104744_3) adjunct to the phthaloyl-CoA decarboxylase (k141_1104744_1) which is differentially expressed. These genes were placed in Bin394 which also contained a differentially expressed benzoyl-CoA reductase subunit C gene (k141_1165338_2). The closest AAI to the Bin394 is *Azoarcus tolulyticus*. The detailed information is listed in **Table S2 and Dataset S1.**

Supplemental References

S1. T. Nishioka, M. Iwata, T. Imaoka, M. Mutoh, Y. Egashira, T. Nishiyama T, T. Shin, and T. Fujii, Appl Environ Microbiol 72:2394–2399, 2006, doi: 10.1128/AEM.72.4.2394-2399.2006.

S2. M. Iwata, T. Imaoka, T. Nishiyama, and T. Fujii, J Biosci Bioeng 122:140–145, 2016, doi: 10.1016/j.jbiosc.2016.01.008.

S3. H. Hara, G. R. Stewart, and W. W. Mohn, Appl Environ Microbiol 76:1516–1523, 2010, doi: 10.1128/AEM.02621-09.

S4. R. Nahurira, L. Ren, J. Song, Y. Jia, J. Wang, S. Fan, H. Wang, and Y. Yan, Curr Microbiol 74:309–319, 2017, doi: 10.1007/s00284-016-1159-9.

S5. S. Fan, J. Wang, K. Li, T. Yang, Y. Jia, B. Zhao, and Y. Yan, J Biotechnol 279:55–60, 2018, doi: 10.1016/j.jbiotec.2018.05.009.

S6. S. Fan, J. Wang, Y. Yan, J. Wang, and Y. Jia, Int J Mol Sci 19:2803, 2018, doi: 10.3390/ijms19092803.

S7. H. Huang, X. Y. Zhang, T. L. Chen, Y. L. Zhao, D. S. Xu, and Y. P. Bai, J Agric Food Chem 67:8548–8558, 2019, doi: 10.1021/acs.jafc.9b02655.

S8. P. Sungkeeree, W. Whangsuk, J. Dubbs, S. Mongkolsuk, and S. Loprasert, Process Biochem 51: 1040–1045, 2016, doi: 10.1016/j.procbio.2016.04.014.

S9. D. K. Hong, S. H. Jang, and C. W. Lee, J Mol Catal B Enzym 133:S337–S345, 2017, doi:10.1016/j.molcatb.2017.02.004.

S10. X. Y. Zhang, X. Fan, Y. J. Qiu, C. Y. Li, S. Xing, Y. T. Zheng, and J. H. Xu, Appl Environ Microbiol 80:6870–6878, 2014, doi: 10.1128/AEM.02072-14.

S11. J. Wu, X. Liao, F. Yu, Z. Wei, and L. Yang, Appl Microbiol Biotechnol 97:2483–2491, 2013, doi: 10.1007/s00253-012-4232-8.

S12. Y. Jiao, X. Chen, X. Wang, X. Liao, L. Xiao, A. Miao, J. Wu, and L. Yang, PLoS One 8:e75977, 2013, doi: 10.1371/journal.pone.0075977.
