# Supplementary material for: Integrated Multi-omics Investigations Reveal the Key Role of Synergistic Microbial Networks in Removing Plasticizer Di-(2-Ethylhexyl) Phthalate from Estuarine Sediments
Source: mSystems. 2021 Jun 8;6(3):e00358-21. doi: 10.1128/mSystems.00358-21 (PMC8269228; doi:10.1128/mSystems.00358-21)
Supplement: TABLE S2 [file msystems.00358-21-st002.pdf]

	Bin97	Bin394
Total length (bp)	3,976,438	6,499,100
# of contigs	528	2633
N50	11547	2890
GC (%)	67.15	67.52
Taxonomy	*Betaproteobacteria*	*Betaproteobacteria*
AAI to closet species (%)	*Thauera aromatica*	*Azoarcus tolulyticus*
CheckM completeness (%)	86.2	79.94
CheckM contamination (%)	14.87	40.83
Phthalate transporter (solute binding)	k141_3776202_21*	Not binned or recovered
Phthalate transporter (fused permease)	k141_3776202_22	Not binned or recovered
phthaloyl-CoA decarboxylase	Not binned or recovered	k141_1104744_1
CoA transferase	Not binned or recovered	k141_1104744_2*
CoA transferase	Not binned or recovered	k141_1104744_3*
Benzoyl-CoA reductase C	Not binned or recovered	k141_1165338_2*

*genes are differentially expressed in phthalic acid-treated mesocosms.
