# Supplementary material for: Integrated Multi-omics Investigations Reveal the Key Role of Synergistic Microbial Networks in Removing Plasticizer Di-(2-Ethylhexyl) Phthalate from Estuarine Sediments
Source: mSystems. 2021 Jun 8;6(3):e00358-21. doi: 10.1128/mSystems.00358-21 (PMC8269228; doi:10.1128/mSystems.00358-21)
Supplement: TABLE S1 [file msystems.00358-21-st001.pdf]

	Accession	Bacterial strain	Substrates	Ref
Actinobacteria	AB214635.1	*Gordonia* sp. P8219	MEHP	[S1]
	LC094142.1	*Rhodococcus* sp. EG-5	MEHP	[S2]
	WP011593638.1	*Rhodococcus* sp. RHA1	MMP, MBP, MHP, MEHP	[S3]
	AMJ52171.1	*Gordonia alkanivorans* YC-RL2	MEHP	[S4]
	AUH67707.1	*Gordonia* sp. YC-JH1	DEHP, DCHP, DOP, DBP	[S5]
	AUH70054.1	*Gordonia* sp. YC-JH1	MBP	[S5]
	MH674097.1	*Gordonia* sp. YC-JH1	MEHP	[S6]
	AYW76487.1	*Gordonia* sp. 5F	DEHP	[S7]
Alphaproteobacteria	KM386872	*Sphingobium* sp. SM42	DBP	[S8]
	AJO67803.1	Sphingobium sp. SM42	DBP	[S8]
	WP_037492848.	S. glacialis PAMC 26605	DBP, DHP, DEP	[S9]
Firmicutes	AEW03609.1	*Sulfobacillus acidophilus* DSM10332	DEP, DPrP, DBP, DPeP, DHP, BBP	[S10]
Gammaproteobacteria	JQ478494.1	*Acinetobacter* sp. M673	DBP, DPP, DPrP, DEP, DHP, DMP	[S11]
Biofilm	KC438416.1	Uncultured bacterium	DPrP, DBP, DPP	[S12]

Abbreviation: benzylbutyl phthalate (BBP), di-n-butyl phthalate (DBP), dicyclohexyl phthalate (DCP), diethyl phthalate (DEP), dihexyl phthalate (DHP), di-n-octyl phthalate (DOP), dipropyl phthalate (DPP), dicyclohexyl phthalate (DCHP), di-(2-ethylhexyl) phthalate (DEHP), dipentyl phthalate (DPeP), diphenyl phthalate (DPhP), Dipropyl phthalate (DPrP), monomethy phthalate (MMP), monobutyl phthalate (MBP), monohexyl phthalate (MHP) and mono-(2-ethylhexyl) phthalate (MEHP).
